# Unravelling the biochemical aspects of the interaction between ticks and *Leishmania* using a tick cell line

**DOI:** 10.1111/mve.70038

**Published:** 2025-12-08

**Authors:** Beatriz Filgueiras Silvestre, Karoline dos Anjos Lima, Fernanda de Paula Pepino, Daniela Cosentino‐Gomes, Adivaldo Fonseca, Lesley Bell‐Sakyi, Georgia Correa Atella, Lucia H. Pinto‐da‐Silva

**Affiliations:** ^1^ Departamento de Microbiologia e Imunologia Veterinária, Instituto de Veterinária, Universidade Federal Rural do Rio de Janeiro Seropédica Rio de Janeiro Brazil; ^2^ Instituto de Bioquímica Médica Leopoldo de Meis, Universidade Federal do Rio de Janeiro Rio de Janeiro Rio de Janeiro Brazil; ^3^ Departamento de Bioquímica Instituto de Química, Universidade Federal Rural do Rio de Janeiro Seropédica Rio de Janeiro Brazil; ^4^ Department of Infection Biology and Microbiomes Institute of Infection, Veterinary and Ecological Sciences, University of Liverpool Liverpool UK

**Keywords:** biochemistry, interaction, ixodidae, *Leishmania*, metabolism, tick cell

## Abstract

Leishmaniasis comprises a group of vector‐borne neglected tropical diseases caused by species of the obligatory intracellular parasite *Leishmania*, transmitted by the bite of dipteran sand flies. Infected dogs serve as the primary domestic reservoir of *Leishmania* parasites and are often found in close association with various arthropods, such as fleas and ticks. There have been recent reports of *Leishmania* infections occurring in areas non‐endemic for sand fly species, leading to reconsideration of the hypothesis that other arthropods, such as ticks, may also play a significant role in the natural history and epidemiology of leishmaniasis. Here, we used a tick cell line as a tool to study *Leishmania infantum* and tick interaction. The results showed that *L. infantum* can bind to and proliferate inside *Ixodes scapularis* IDE8 tick cells. The infection did reduce tick cell viability and induce reactive oxygen species (ROS) production. Lipid profile analysis showed that the presence of *L. infantum* increased oxysterol in tick cells and influenced tick cell lipid biosynthesis, since an increase in glycerolipids and esterified cholesterol was observed in infected cells at 48 h. Further experiments are necessary to elucidate whether *Leishmania* can overcome the various biochemical and tissue barriers within ticks and be transmitted to the host.

## Graphical abstract

- *Leishmania infantum* propagated in IDE8 tick cell line in vitro.
- *L. infantum* reduces tick cell viability and induces reactive oxygen species production.
- Lipidic profile of IDE8 tick cell line is altered during *Leishmania* infection.

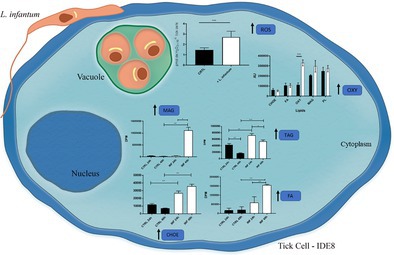

## INTRODUCTION

Leishmaniasis encompasses a group of vector‐borne neglected tropical diseases caused by the obligate intracellular parasite *Leishmania*. Female sand flies, mainly *Phlebotomus* spp. and *Lutzomyia* spp., serve as natural vectors, transmitting the parasite to mammals, including humans and dogs. However, there have been recent reports of *Leishmania* infections occurring in areas non‐endemic for sand fly species, leading to the reconsideration of an old hypothesis that other arthropods, such as culicides, midges, fleas and ticks, may also play a significant role in the natural history and epidemiology of leishmaniasis (Dantas‐Torres, 2006; Dougall et al., 2011; Kaewmee et al., 2023; Otranto & Dantas‐Torres, 2010; Rojas‐Jaimes et al., 2022; Slama et al., 2014).

Infected dogs serve as the primary domestic reservoir of *Leishmania* parasites and are often found in impoverished peri‐domestic areas that are infested by ectoparasites, including ticks. Several studies have detected *Leishmania* DNA in different tick organs, including the salivary glands, midgut and ovaries (Rojas‐Jaimes et al., 2022; Viol et al., 2016). *Leishmania* kinetoplast DNA has also been found in naïve ticks at different stages of development after feeding on an infected dog, indicating both interstadial and transovarial transmission of the parasite (Dabaghmanesh et al., 2016). However, despite detecting *Leishmania* DNA in ticks fed on infected dogs using PCR assay, one study on the survival of the parasites in the ticks showed no *Leishmania* growth in a culture medium (Paz et al., 2010). Recently, *Rhipicephalus sanguineus* infected with *Leishmania* by artificial feeding showed an increase in haemocyte and phenoloxidase activity, as well as the presence of *Leishmania infantum* DNA until 7 days post‐infection (Feitosa et al., 2018).

Recent studies have made significant strides in elucidating the biology underpinning tick vectorial ability through different approaches, such as immunology, biochemistry and entomological analysis. Research has also demonstrated the influence of pathogens on vector metabolism, such as lipid metabolism, oxidative phosphorylation and glycolysis, and how these factors affect vector competence considering immune and stress responses (Buysse et al., 2021; de la Fuente et al., 2017; Jia et al., 2020; Samaddar et al., 2020). The development of tick cell lines has proved to be a useful tool for advancing research on various tick and tick‐borne diseases, helping to clarify biological, biochemical and molecular aspects of pathogen–tick interactions (Bell‐Sakyi et al., 2007, 2018). Although *L. infantum* is most often found in *R. sanguineus*, the presence of *Leishmania* was also identified in *Ixodes ricinus* in Italy (Magri et al., 2022). Considering the availability of a well‐established tick cell line IDE8, derived from *Ixodes scapularis*, which belongs to the familyIxodidae (Munderloh et al., 1994), we decided to employ it as a conceptual model to reveal biological aspects of *Leishmania* and tick cell interactions and thereby provide a better understanding of this relationship.

## MATERIALS AND METHODS

### Parasite

*Leishmania infantum* (MCAN/BR/2008/1112), originally isolated in 2008 from a dog in Brazil, was maintained in Schneider's insect medium supplemented with 10% foetal bovine serum (FBS) and 50 μg/mL gentamicin (Sigma) at 26°C or Schneider's insect medium supplemented with 10% FBS, 2% human urine and 50 μg/mL gentamicin (Sigma) at 26°C, in sealed T25 tissue culture flasks with weekly subculture.

### Tick cell line

The *I. scapularis* tick cell line IDE8 was cultured in sealed T25 flasks in 5 mL of complete L15B medium as described previously (Marotta et al., 2018), at 32°C.

### Leishmania*—tick cell interaction in vitro*

IDE8 tick cells (2 × 10^5^ per well) were seeded onto round glass coverslips in 24‐well plates and maintained at 34°C overnight. After this period, the cells were incubated with *L. infantum* promastigotes at a multiplicity of infection of five parasites to one cell (MOI 5) for 2 h. Following the incubation, free parasites were removed by washing with phosphate‐buffered saline (PBS), and the interaction was assessed at 2, 24 and 48 h. At the end of each time period, the coverslips were fixed in methanol and stained with Giemsa or Diff‐Quick. The association index (% infected cells × number of parasites/cell) was determined by counting at least 200 cells per coverslip. The data represent three independent experiments in triplicate.

At 48 h post inoculation, *Leishmania* infected‐IDE8 tick cells were incubated with Schneider's insect medium and incubated at 27°C for more than 48 h, to allow viable parasites inside the cells to grow. Then, the number of promastigotes recovered was counted in a Neubauer chamber.

### Lactate dehydrogenase activity

To evaluate tick cell viability during *Leishmania* interaction, IDE8 tick cells (2 × 10^5^ per well of a 24‐well plate) were incubated with or without *L. infantum* promastigotes (MOI 5) for 2, 24 or 48 h at 34°C, as described above. Then, 50 μL of supernatant was used to evaluate lactate dehydrogenase (LDH) activity using a CytoTox96 Non‐Radioactive Cytotoxicity Assay kit (Promega). LDH activity was read at 490 nm at 23°C using a SpectraMax spectrophotometer (Molecular Devices). The data represent 2 independent experiments in triplicate.

### Reactive oxygen species production by tick cells

To measure reactive oxygen species (ROS) production by tick cells during *Leishmania* interaction, IDE8 tick cells (2 × 10^5^ per well of a 24‐well plate) were incubated with or without *L. infantum* promastigotes (MOI 5) at 34°C for 2 h. Non‐adherent parasites were removed by washing and the cells were incubated at 34°C. After 48 h, tick cells were washed with PBS, counted and assayed for ROS production. Extracellular hydrogen peroxide (H_2_O_2_) production was quantified by the Amplex Red oxidation method (Invitrogen®). Tick cells were added to a reaction medium containing PBS, 10‐μM Amplex Red and 0.1 U/mL horseradish peroxidase (HRP) in a final volume of 0.2 mL at room temperature. Reactions without cells were considered as blanks, and uninfected cells were used as controls. After 1 h of reaction, resorufin formation was measured by the change in absorbance at 540 nm (Rocco‐Machado et al., 2019). The data represent 2 independent experiments in quadruplicate.

### Lipid extraction

IDE8 tick cells (1 × 10^6^/mL in T25 flasks) were incubated with or without *L. infantum* promastigotes (MOI 5) for 48 h at 34°C, as described above. After this period, tick cells were washed three times with PBS and used for lipid extraction, which was performed according to Bligh and Dyer (1959). A mixture of methanol:chloroform:H_2_O (2:1:0.8 v/v) was added to the samples. After intermittent agitation for 2 h, the solution was centrifuged for 20 min at 3300 × g in a clinical centrifuge and the supernatant was collected. The precipitate was subjected to a second extraction with the same mixture, followed by intermittent agitation for 1 h, and centrifugation for 20 min at 2000 × g. The supernatants were pooled, and 1.0 mL of water and 1.0 mL of chloroform were added thereto. After stirring and verifying the presence of two phases, the material was centrifuged again for 30 min at 3300 × g. The organic phase (lower), containing the lipids, was then removed with the aid of a Pasteur pipette and stored at −4°C.

### High‐performance thin layer chromatography

Extracted lipids were analysed by high‐performance thin layer chromatography (HPTLC), as described previously for neutral lipids (Kawooya et al., 1988) and phospholipids (PL) (Horwitz & Perlman, 1987). Each lipid spot was identified by comparison with lipid standards run in parallel. Aliquots of 5 μg each of 1‐oleoyl‐rac‐glycerol (MG), 1,3‐diolein (DAG), glycerol trioleate (TAG), cholesterol (CHO), cholesteryl palmitate (CHOE), oleic acid (FA), 24‐hydroxycholesterol (oxysterol—OXY) were used as the lipid standards, purchased from Sigma‐Aldrich® (St Louis, Missouri, USA). To visualize the lipids, plates were immersed in a carbonization solution consisting of 8% CuSO_4_ and 10% H_3_PO_4_ for 10 s and heated at 110°C for 20 min. Plates were analysed by densitometry using ImageMaster TotalLab software (TotalLab, Newcastle, UK). The data represent three independent experiments.

### Measurement of uptake of ^3^H‐palmitic acid precursor

IDE8 tick cells (1 × 10^6^/mL in T25 flasks) were incubated with or without *L. infantum* promastigotes (MOI 5) at 34°C as described above. Cells were incubated with 100 μCi of ^3^H‐palmitate (^3^H‐palmitic acid 16:0 [9.10‐3H(N)]) (PerkinElmer, Boston, MA) complexed with 100 microliter of fatty acid‐free albumin (BSA‐FFA, Sigma‐Aldrich®, St Louis, Missouri, USA). After 48 h of interaction, cells were subjected to lipid extraction and HPTLC. The lipid spots were scraped off the silica sheet, and radioactivity associated with each lipid was determined by scintillation counting using a PerkinElmer TriCarb scintillator. The data represent four independent experiments.

### Statistical analysis

The data were analysed using Student's t‐test to compare two groups and analysis of variance (ANOVA) for more than two groups. Analyses were performed using GraphPad Prism 8.0 software. Statistical differences were considered significant when *p* ≤ 0.05.

## RESULTS

**FIGURE 1:**
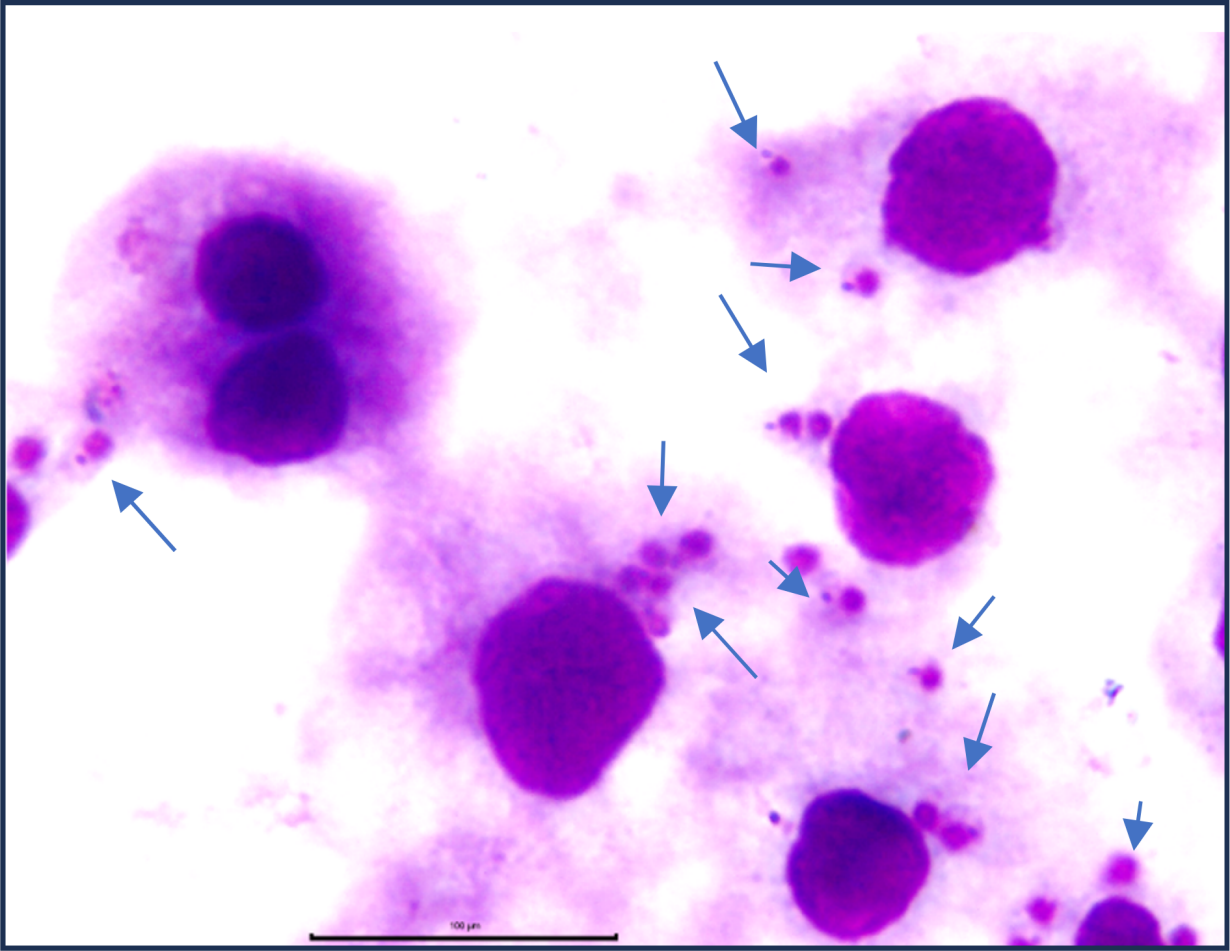
IDE8 tick cells were seeded onto coverslips and then incubated with *Leishmania infantum* promastigotes (cell:parasite ratio of 1:5) at 34°C for 48 h. Then, cells were fixed with methanol and stained with Diff‐Quick. Arrows indicate the parasites. Bars = 10 μm.

**FIGURE 2:**
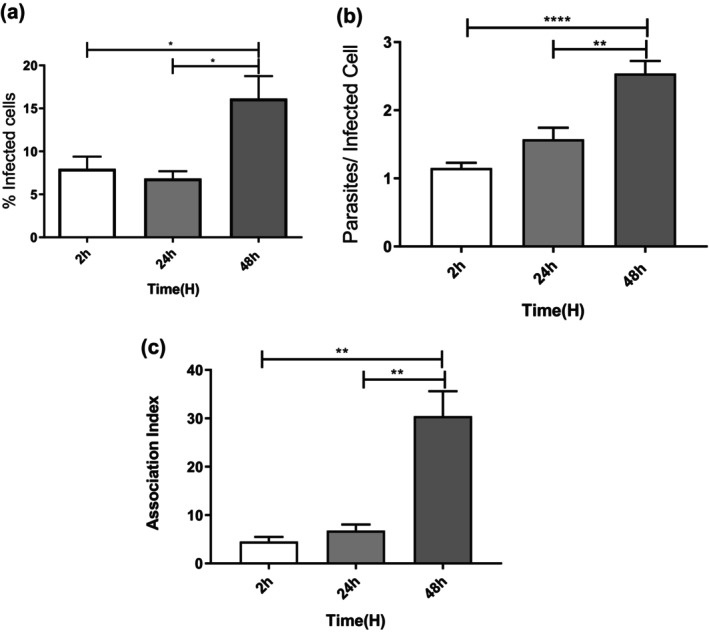
Interaction between IDE8 tick cells and *Leishmania infantum*. IDE8 cells were incubated with *L. infantum* promastigotes at MOI 5. After 2 h, IDE8 cells were washed with phosphate‐buffered saline (PBS) to remove free parasites and were incubated for the indicated times. After these time periods, (a) % infected cells, (b) number of parasites per infected cell and (c) association index were determined. Data = mean ± SEM of three experiments in triplicate. *p < 0.0254; **p < 0.0017; and ****p < 0.0001.

First, to determine the capacity of *L. infantum* promastigotes to interact with *I. scapularis* IDE8 cells, the parasites were incubated with tick cells at 2, 24 or 48 h and the presence of intracellular parasites was evaluated (Figure 1). At 2 h post‐inoculation, 8% of tick cells contained parasites, falling to 6.8% at 24 h. At 48 h, there was a twofold increase in the percentage of *L. infantum*‐infected tick cells compared to 2 h (Figure 2a). The number of parasites per infected IDE8 cell analyses showed no significant difference between 2 h (mean 1.2 parasites per cell) and 24 h (mean 1.5 parasites per cell). At 48 h post‐interaction, however, the number of parasites per infected cell more than doubled compared to the number observed at 2 h (Figure 2b). The association index at 48 h indicated a sevenfold increase compared to 2 h and a 4.5‐fold increase compared to 24 h (Figure 2c). It was also observed that after 48 h of *Leishmania* interaction with IDE8 tick cells at 34°C, it was possible to recover 5 × 10^6^ promastigotes/mL in the supernatant (data not shown).

**FIGURE 3:**
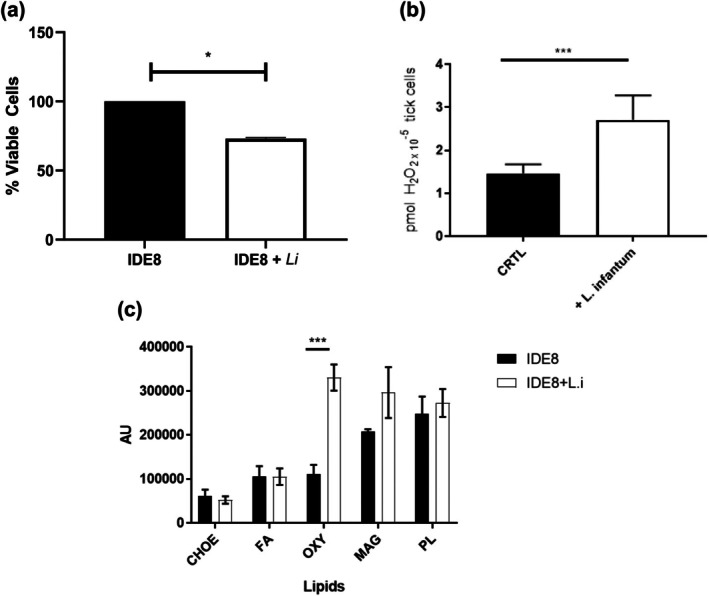
IDE8 tick cell response to the presence of *Leishmania infantum*. IDE8 cells were incubated with or without *L. infantum* (*L.i*.) promastigotes (MOI 5) for 2 h. Then, the cells were washed with phosphate‐buffered saline (PBS) and incubated for 48 h at 34°C. (a) The supernatant was collected, and lactate dehydrogenase (LDH) activity was read using a spectrophotometer. The percentage of viable cells were calculated considering uninfected cells as control (100%). Data = mean ± SEM from 2 independent experiments in triplicate. *p > 0.0001. (b) The cells were then incubated with amplex red reagent and H_2_O_2_ production was read at 540 nm. Data = media ± SEM from 2 independent experiments in quadruplicate. ***p = 0.0003. (c) After that, the lipids were extracted and analysed by high‐performance thin layer chromatography. Data = media ± SEM of three independent experiments. AU, arbitrary unit; CHOE, esterified cholesterol; FA, fatty acid; MAG, monoacylglycerol; OXY, oxysterol; PL, phospholipid. ***p = 0.0002.

Evaluation of IDE8 cell viability by measurement of LDH activity at 48 h post‐infection showed a reduction of 30% of viability in *L. infantum*‐infected IDE8 cells compared to uninfected cells (Figure 3a).

To investigate if ROS could be involved in tick cell–parasite interaction, we measured the generation of H_2_O_2_ 48 h after infection. Our data showed that IDE8 cells were able to generate ROS in response to *L. infantum* interaction. The results showed a twofold increase in the H_2_O_2_ level in *L. infantum*‐infected IDE8 cells compared to uninfected cells (Figure 3b).

**FIGURE 4:**
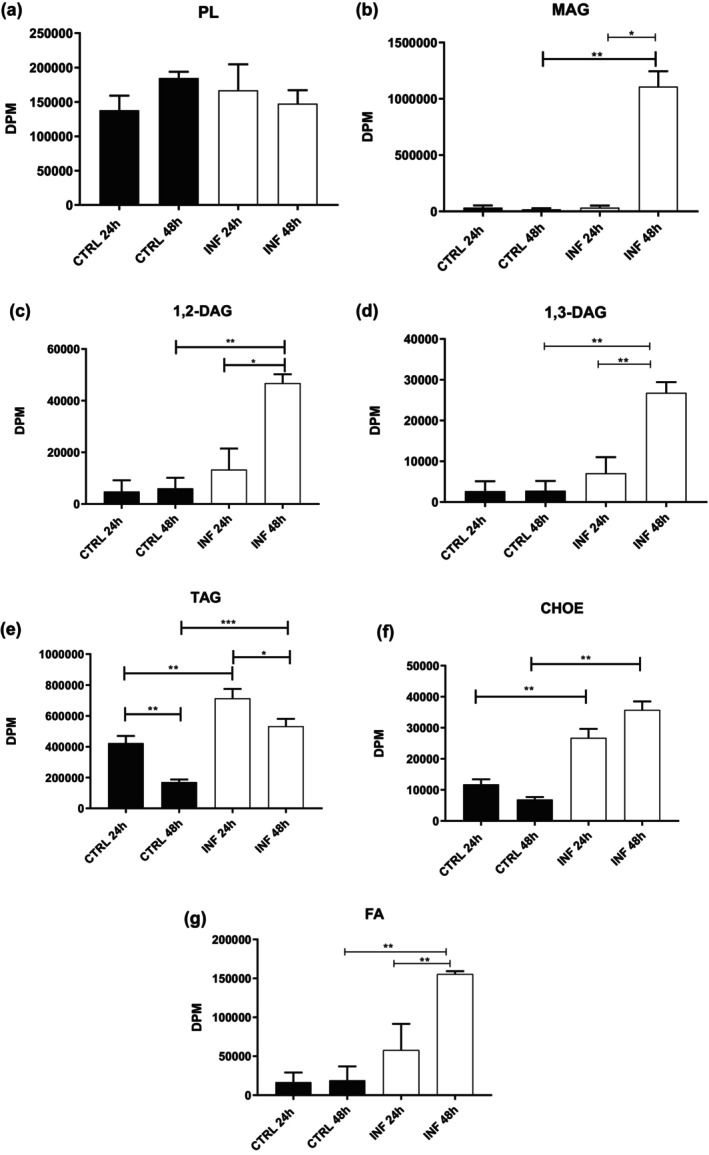
*Leishmania* modulates tick cell lipid metabolism. IDE8 cells were incubated with (INF) or without (CTRL) *Leishmania infantum* promastigotes at MOI 5, in the presence of 5 μCi of ^3^H‐FA‐BSA. The lipids were extracted, and the samples were analysed at the indicated times. Results are shown for (a) Phospholipid (PL), (b) monoacylglycerol (MAG), (c) 1,2‐diacylglycerol (1,2‐DAG), (d) 1,3‐diacylglycerol (1,3‐DAG), (e) triacylglycerol (TAG), (f) esterified cholesterol (CHOE) and (g) fatty acids (FA). Values = mean + SEM of four independent experiments. DPM, disintegrations per minute. *p < 0.04, **p < 0.007, ***p < 0.002.

Analysis of the profiles of five neutral lipids of *L. infantum*‐infected and uninfected IDE8 cells showed a threefold increase in oxysterol (OXY) in infected tick cells compared to uninfected cells, while differences in the other four lipids were not significant (Figure 3c). A new assay was then designed to understand whether there was a difference in the uptake of palmitic acid, a precursor used to synthesize neutral lipids and/or in the lipid metabolism of uninfected versus *L. infantum*‐infected tick cells incubated with radioactive palmitic acid. The results showed no significant difference in PL between infected and uninfected IDE8 cells (Figure 4a). In contrast, there was a significant (40‐fold) increase in the amount of monoalcylglycerol (MAG) found in infected IDE8 cells compared to uninfected control cells at 48 h (Figure 4b). Comparing infected IDE8 cells over time, a 24‐fold increase was observed in the amount of MAG at 48 h post‐infection compared to 24 h post‐infection (Figure 4b). Subsequently, we evaluated the amount of 1,2‐D, 1,3‐D and FA lipids, which were higher in *L. infantum*‐infected IDE8 cells at 48 h post‐infection compared to uninfected tick cells (Figure 4c,d,g). Analysis of TAG demonstrated a 2.6‐fold decrease between 24 h and 48 h in uninfected control IDE8 cells (Figure 4e). A similar profile was observed in infected tick cells, which showed a twofold reduction from 24 h to 48 h. However, at both time points the TAG levels were significantly higher in *L. infantum‐*infected IDE8 cells compared to uninfected control cells (Figure 4e). CHOE analysis showed almost twofold and threefold increases in the amount of CHOE in *L. infantum*‐infected IDE8 tick cells at, respectively, 24 h and 48 h post‐infection compared to uninfected cells (Figure 4f).

## DISCUSSION

The involvement of other arthropods (ticks, fleas, culicides and midges), besides sandflies, as biological or mechanical vectors of *Leishmania* parasites, especially in cases where Phlebotominae are not present, has been a subject of long‐standing debate (Blanc & Caminopetros, 1930; Dantas‐Torres, 2008; Dougall et al., 2011; Giraud et al., 1950; Kaewmee et al., 2023; Slama et al., 2014). In our study, we demonstrated the ability of *Leishmania* to interact with tick cells using an *I. scapularis* cell line. Our results revealed that *Leishmania* can interact with IDE8 tick cells and remain within them for at least 48 h. Similar to our results, other ixodid tick cell lines, such as *Rhipicephalus appendiculatus* and *Rhipicephalus evertsi* embryo‐derived cell lines infected with *Leishmania donovani* and *Leishmania major* also showed an increase in the number of infected cells after 48 h of incubation and the use of tick cells to propagate and cultivate *Leishmania* in vitro was proposed (Nyindo et al., 1987). However, the present study uniquely contributes to the broader understanding of *Leishmania*‐tick interactions by demonstrating that *L. infantum* can bind, invade and persist in *I. scapularis*‐derived IDE8 cells for 48 h. The choice of IDE8 tick cells as a proof of concept was realized since they belong to the family Ixodidae, and although *Leishmania* parasites have not been detected in *I. scapularis* until now, these results showed that *Leishmania* parasites propagated inside these tick cells. Moreover, in a recent study the presence of *Leishmania* was detected in *I. ricinus* in a rural area of north‐eastern Italy, where foci of visceral human leishmaniasis were identified (Magri et al., 2022). Although these findings provide valuable information in early events of interaction and are important indicators of active host–parasite interplay, the limited observation period of 48 h prevents conclusions on longer‐term outcomes such as sustained parasite replication, adaptation to different niches, immune evasion strategies or survival across tick developmental stages. Added to that, the importance of different tick cell lines as a tool to unravel aspects of pathogens and ticks has been discussed (Salata et al., 2021).

Therefore, the data confirm early events of interaction; they do not establish whether ticks could function as competent vectors, a question that requires further in vitro and in vivo analysis. In vivo analysis of *R. sanguineus* infected with *Leishmania* has shown the presence of parasites in different tick organs, such as ovaries, midgut and salivary glands, suggesting that *Leishmania* could be circulating inside the tick (Viol et al., 2016) Our results also showed that the presence of *Leishmania* reduced IDE8 tick cell survival by 30%, indicating an alteration to tick cell viability in vitro. If *Leishmania* parasites could in fact remain in ticks and be transmitted, it needs to be elucidated through in vivo experiments.

Among all ROS molecules, H_2_O_2_ is a major redox metabolite operator in redox sensing, signalling and redox regulation (Sies, 2017). The increase in H_2_O_2_ production during insect infection has been reported as a protective mechanism of the immune system to limit the development of parasites in the host (Hao et al., 2003; MacLeod et al., 2007; Souza et al., 1997). In different *Anopheles gambiae* strains, the higher the systemic H_2_O_2_ levels, the better the mosquitoes survived a bacterial or malaria parasite challenge (Kumar et al., 2003; MacLeod et al., 2007; Molina‐Cruz et al., 2008; Souza et al., 1997). H_2_O_2_ generation was also shown to be at higher levels in *Aedes aegypti* infected with the trypanosomatid *Strigomonas culicis* (Bombaça et al., 2021), during bacterial infection in the gut of *Ctenocephalides felis* cat fleas (Brown et al., 2021) and in *Rhodnius prolixus* infected with *Trypanosoma rangeli* (Cosentino‐Gomes et al., 2014). Our findings indicate that IDE8 tick cells produce H_2_O_2_ in response to *L. infantum*. Interestingly, a recent study showed that in vivo infection of *R. sanguineus* with *L. infantum* promastigotes induced an increase in nitric oxide (NO) production and phenoloxidase (PO) activity in tick haemolymph. Coincidentally, the increase in PO activity correlated with the presence of a high number of parasites (Feitosa et al., 2018). Either H_2_O_2_ or NO could play a signalling role for the activation of the tick immune system, as shown for *Anopheles albimanus* infected with *Plasmodium berghei* (Herrera‐Ortiz et al., 2011).

Studies investigating how pathogens interact with arthropods and their lipid metabolism have been conducted using various models (O'Neal et al., 2020). For example, models of *Ae. aegypti*–*Plasmodium gallinaceum* and *R. prolixus*–*T. rangeli* have demonstrated alterations in the insects lipoproteins. In an *Aedes albopictus* cell line infected with dengue virus, fatty acid synthase activation resulted in increased biosynthesis of PL, sphingolipids and TAG. In the present study, lipid analysis showed a significant increase in the levels of oxysterol—a lipid derived from cholesterol through a few pathways, including non‐enzymatic oxidation—in *L. infantum*‐infected IDE8 cells. In this pathway, cholesterol is easily oxidized to oxysterol in the presence of ROS, representing the first step of lipid peroxidation, a process considered an ‘antioxidant’ cholesterol pathway by some authors (Girão et al., 1999). In mammals, oxysterol has been observed to have several biological effects, including cytotoxicity and antiproliferative activity in tumour cells (Freitas et al., 2021). Furthermore, oxysterol regulates cholesterol homeostasis by modulating the activity of two transcription factors: sterol regulatory element binding proteins (SREBP) and liver X receptors (LXR). SREBP inhibition regulates fatty acid and cholesterol biosynthesis, as well as cholesterol uptake, exerting an inhibitory effect on hydroxymethyl glutaryl coenzyme A (HMG‐CoA), an enzyme vital for cholesterol biosynthesis. LXR activation induces cholesterol efflux and fatty acid synthesis. Thus, high levels of oxysterol in the mammalian body, due to a large accumulation of cholesterol, inhibit its uptake while promoting its removal from the cell (Choi & Finlay, 2020; Wang et al., 2008). Moreover, oxysterol has already been discussed as an important immunomodulator in mammals, as its addition to compounds with leishmanicidal activity increased the inhibition of *L. donovani* growth and induced parasite death in vitro, even in strains modified to be resistant to amphotericin B (Bazin et al., 2006). Our data suggest that the presence of the parasite caused an increase in oxysterol in tick cells, which could indicate a regulatory response to parasites. Additionally, this increase was observed after 48 h, coinciding with the increase in ROS production. This may indicate a preference for the non‐enzymatic pathway of oxysterol formation. Unfortunately, it was not possible to investigate whether there was any difference in the amount of total cholesterol in the samples; this topic is reserved for future research.

For the first time, the lipid biosynthesis analysis revealed modulation of glycerolipids in *L. infantum*‐infected tick cells compared to uninfected cells. Within 24 h, both infected and uninfected IDE8 cells presented a large amount of TAG, which decreased by 48 h. Arthropods are known to store large amounts of energy in the form of TAG in various organs, including cellular structures called lipid droplets (Soulages et al., 2015). Some mammalian cells, such as bone marrow‐derived macrophages and dendritic cells, have shown increased TAG synthesis when infected with *Leishmania* spp. (Lecoeur et al., 2013; Rabhi et al., 2016). Similarly, a change in the lipidic profile of insect cells with an accumulation of various lipids, particularly PL, was observed in the model of infection of *Ae. albopictus* cells with Zika virus (Gondim et al., 2018). Interestingly, our data suggest that both uninfected and *L. infantum*‐infected tick cells experienced a reduction in TAG from 24 to 48 h. However, even with this decrease, TAG content was higher in infected IDE8 cells than in uninfected cells. Concurrently, a significant increase was observed in these cells of all metabolites of the TAG degradation pathway, including MAG, 1,2‐DAG, 1,3‐DAG and free fatty acids. This suggests that the tick cells may have recruited energy molecules to help control *L. infantum* infection. Alternatively, the parasite could be modulating lipid metabolism in the tick cell line, consuming the TAG synthesized by the cell. In the latter scenario, the host cell would be synthesizing the necessary lipid precursors to replenish its energy store. To elucidate these mechanisms, further studies could evaluate the expression of the enzymes involved in the TAG synthesis and degradation pathway or measure the activity of these enzymes.

Furthermore, we observed an increase in CHOE levels in *L. infantum*‐infected IDE8 cells up to 48 h. Cholesterol is esterified to CHOE through the enzyme cholesterol acetyltransferase (ACAT). Some studies have reported that different pathogens can alter the cholesterol pathway in their vectors/hosts, although only two studies have observed changes in the lipid profile of ticks in vivo (Hoxmeier et al., 2017; Sá et al., 2018). In both of these studies, the absence of microorganisms led to an increase in cholesterol levels. In another study, the *I. scapularis* cell line ISE6 was infected with Langat virus; although *I. scapularis* is not its natural vector, this less virulent flavivirus was used as an in vitro infection model for highly pathogenic tick‐borne flaviviruses. The data showed that several proteins related to tick cell metabolism were altered during infection, including ACAT1, which uses coenzyme A (CoA) metabolites during fatty acid metabolism, and VNN, a protein anchored by glycosylphosphatidylinositol involved in lipid remodelling. This suggests that decreased expression of these proteins reduces viral replication (Grabowski et al., 2017).

In our results, the observed metabolic shifts—such as oxysterol accumulation and TAG degradation—mirror responses seen in other vector‐pathogen systems, suggesting that *Leishmania* may exploit tick lipid metabolism for survival. However, it remains to be seen whether *Leishmania* could in fact overcome the various tissue and biochemical barriers within ticks and be transmitted to the host during feeding. Pathogens can potentially influence vector metabolism, and the transmission ability of vectors has been correlated with their pathogen tolerance or elimination response (Samaddar et al., 2020).

## CONCLUSIONS

The present study uniquely contributes to the broader understanding of *Leishmania*‐tick interactions by demonstrating that *L. infantum* not only invades *I. scapularis*‐derived IDE8 cells but also alters their metabolic and oxidative profiles within just 48 h. Despite the relatively short period of analysis, our findings show active parasite survival and proliferation, measurable ROS response and significant lipid remodelling in infected cells—critical early indicators of host‐pathogen interaction. These results, although limited to in vitro conditions, reinforce the plausibility of ticks playing a biological or mechanical role in *Leishmania* transmission, especially in non‐endemic areas where sand flies are absent but infected dogs and ectoparasites are prevalent. Moreover, they reinforce the use of tick cell lines as convenient, affordable and valuable tools in the search for a deeper understanding of the biochemical aspects of vector‐pathogen interactions, paving the way for more targeted in vivo studies.

## AUTHOR CONTRIBUTIONS

**Beatriz Filgueiras Silvestre:** Methodology; conceptualization; writing – review and editing; investigation. **Karoline dos Anjos Lima:** Methodology; writing – review and editing; software. **Fernanda de Paula Pepino:** Methodology. **Daniela Cosentino‐Gomes:** Methodology; writing – review and editing; formal analysis. **Adivaldo Fonseca:** Conceptualization; writing – review and editing; writing – original draft. **Lesley Bell‐Sakyi:** Writing – review and editing; writing – original draft. **Georgia Correa Atella:** Methodology; writing – original draft. **Lucia H. Pinto‐da‐Silva:** Conceptualization; writing – original draft; writing – review and editing; funding acquisition; resources; supervision; formal analysis; methodology; validation; visualization; investigation; data curation; project administration.

## FUNDING INFORMATION

We received financial fellowship support from Fundação de Amparo à Pesquisa do Estado do Rio de Janeiro (FAPERJ) and Conselho Nacional de Desenvolvimento Científico e Tecnológico (CNPq) for B.F.S. L.B.S. was funded by the Wellcome Trust grant no. 223743/Z/21/Z.

## CONFLICT OF INTEREST STATEMENT

The authors declare no conflicts of interest.

## Data Availability

All the datasets supporting the conclusions of this article are included within the article. Filgueiras et al. (2025). Data from: Unravelling the biochemical aspects of the interaction between ticks and *Leishmania* using a tick cell line [Dataset]. Dryad. https://doi.org/10.5061/dryad.dv41ns2bb.
